# Thompson Sampling for Device-to-Device Relay Selection Under a 3GPP-Aligned mmWave Channel Model: A Bandit-Theoretic Study with Bernoulli and Gaussian Variants

**DOI:** 10.3390/s26217069

**Published:** 2026-11-09

**Authors:** Ali Nauman, Moin Ali, Sung Won Kim

**Affiliations:** School of Computer Science and Engineering, College of Digital Convergence, Yeungnam University, Gyeongsan-si 38541, Gyeongbuk-do, Republic of Korea; moinali@yu.ac.kr (M.A.); swon@yu.ac.kr (S.W.K.)

**Keywords:** device-to-device (D2D) communication, relay selection, Thompson Sampling (TS), multi-armed bandit (MAB), millimeter-wave (mmWave)

## Abstract

Device-to-Device (D2D) communication is a key technology for 5G-Advanced and 6G networks, enabling nearby User Equipment (UE) to communicate directly or via a relay when the direct link is blocked or unreliable. At Millimeter-Wave (mmWave) frequencies, frequent blockage and intermittent connectivity make relay selection a sequential decision problem under uncertainty, naturally formulated as a Multi-Armed Bandit (MAB). This paper develops and evaluates two Thompson Sampling (TS) approaches for D2D relay selection using a 3GPP TR 38.901 UMi-Street Canyon channel model. The first TS approach uses a Beta-Bernoulli model to maximize link-success probability, whereas the second TS uses a Gaussian-conjugate model to maximize continuous spectral efficiency. Over T=2000 rounds with N=10 candidate relays and shared channel realizations, both approaches identify the best relay within about 100–150 rounds and direct more than 94% of selections to it. Compared with random selection, they raise the mean spectral efficiency by ≈3×, raise the link-success rate to above 99%, and cut the outage probability to 0.9% (TS-Bernoulli) and 0.4% (TS-Gaussian). These results demonstrate the effectiveness of TS for relay selection under realistic mmWave propagation conditions and highlight the importance of aligning the learning objective with the target performance metric.

## 1. Introduction

The proliferation of proximity-based mobile services, including local content sharing, vehicle-to-vehicle safety messaging, augmented-reality overlays, and public-safety mesh communications, has made Device-to-Device (D2D) communication a standard component of the cellular air-interface roadmap from 5G through 5G-Advanced and into the early 6G era [1,2]. D2D communication enables two nearby user equipment units to exchange data directly, bypassing the Base Station (BS)/(gNB) for the payload path. This approach reduces end-to-end latency, offloads the radio access network, and can substantially increase spatial spectral reuse. When a direct link between a source User Equipment (UE) and a destination UE is unavailable due to blockage, or because regulatory or interference constraints prevent direct transmission, a third UE can act as a decode-and-forward or amplify-and-forward relay [3,4,5]. This extends coverage and restores connectivity at the cost of an additional hop.

The choice of which UE should serve as the relay is far from trivial. In a typical urban microcell (UMi) scenario, there may be anywhere from a handful to several dozen candidate relay UEs in the vicinity of the source, each experiencing a distinct, time-varying, and a priori unknown channel to both the source and, implicitly, the eventual destination [6,7]. The channel quality of each candidate is influenced by distance-dependent path loss, the presence or absence of a Line-of-Sight (LoS) path, log-normal shadow fading, and fast time-scale small-scale fading. This challenge becomes particularly acute at Millimeter-Wave (mmWave) frequencies, which are central to current 5G New Radio (NR) FR2 deployments and to the sub-THz research direction actively being pursued for 6G [8]. At these frequencies, path loss is severe and diffraction is weak; consequently, even modest obstacles, such as a pedestrian body, a passing vehicle, or foliage, can introduce tens of decibels of additional attenuation by causing a link to transition from LoS to Non-Line-of-sight (NLoS) propagation. Relaying therefore represents, in a very concrete sense, one of the key techniques proposed in the 6G research community to mitigate the effects of mmWave and sub-THz blockage, whether through opportunistic UE-to-UE relaying, Reconfigurable Intelligent Surfaces (RISs), or dedicated relay nodes [9].

Relay selection under these conditions can naturally be formulated as a sequential decision-making problem under uncertainty [10]. A scheduler—which may reside at the source UE, within a dedicated relay-selection function, or at the serving gNB—must repeatedly select one relay from a set of candidates, observe a measure of the resulting link quality, and use this feedback to inform subsequent selections, all without being given an explicit characterization of which relay is optimal. This is precisely the structure of the classical Multi-Armed Bandit (MAB) problem: a fixed set of arms, each associated with an unknown reward distribution, is selected sequentially with the objective of maximizing cumulative reward, or, equivalently, of minimizing regret relative to the best arm in hindsight [11]. The MAB formulation is particularly well suited to relay selection because it does not require prior channel-state information, a separate training phase, or an explicit channel model at run time. Instead, it learns the relative quality of candidate relays directly from the sequence of observed outcomes. This is consistent with the information available to a practical D2D scheduler, which can obtain link-quality feedback through mechanisms such as sounding reference signals, discovery beacons, or Hybrid Automatic Repeat Request (HARQ) feedback.

Among MAB algorithms, Thompson Sampling (TS)—a Bayesian approach dating back to Thompson’s 1933 work on sequential clinical trial allocation [12]—has become one of the most widely used and well-studied methods, owing to its strong empirical performance [13], near-optimal theoretical regret guarantees [14,15], and conceptual simplicity. At each round, TS samples a plausible reward for each arm from that arm’s current posterior distribution and selects the arm with the highest sampled reward. This mechanism naturally balances exploration and exploitation without requiring a hand-tuned exploration schedule, in contrast to simpler heuristics such as ε-greedy selection.

This paper presents a study of TS-based D2D relay selection under a channel model aligned with 3GPP Technical Report (TR) 38.901 [16], the standard reference channel model used in 3GPP NR system-level studies over the frequency range 0.5 to 100 GHz. We deliberately consider the Urban Microcell Street Canyon (UMi-SC) scenario at a carrier frequency of 28 GHz, both because it is representative of currently deployed mmWave spectrum supporting D2D communication (e.g., NR band n257) and because its blockage-prone, short-range propagation characteristics make it a relevant regime for studying relay selection and closely align with the focus of recent 6G relaying research. This paper makes the following contributions:

1. A 3GPP-aligned channel model. We employ the full 3GPP TR 38.901 UMi-SC channel model, including the breakpoint-distance-dependent LoS/NLoS path-loss formulation, distance-dependent LoS probability, LoS/NLoS-specific log-normal shadow-fading standard deviations, and Rician (LoS) and Rayleigh (NLoS) small-scale fading with a physically motivated Rician *K*-factor.
2. Two TS variants with distinct, well-defined objectives. We formulate and implement both a Beta-Bernoulli TS bandit that directly targets link-success probability (and hence outage minimization) and a Gaussian-conjugate TS bandit that directly targets continuous spectral efficiency (and hence throughput maximization). We demonstrate that these represent genuinely different optimization objectives and therefore should not be conflated when evaluating bandit algorithms in a communications context.
3. A methodologically fair, shared-randomness comparison protocol. All algorithms under comparison are evaluated using identical per-round channel realizations, which are generated once and shared across algorithms. This ensures that observed performance differences are attributable to the algorithms’ decision policies rather than to favorable or unfavorable channel realizations.
4. A multi-dimensional evaluation suite. We evaluate performance using complementary metrics, including cumulative average reward, cumulative regret relative to a reward oracle, moving-average link-success rate, relay-selection distribution, and the empirical Cumulative Distribution Function (CDF) of the achieved Signal-to-Interference-plus-Noise Ratio (SINR), together with the corresponding outage-probability readout. This combination provides a more comprehensive characterization of algorithm behavior than any single summary statistic, capturing both average performance and important behavioral and tail characteristics, such as relay-selection preferences and outage risk.

Existing D2D relay-selection schemes are model-based: they evaluate a selection rule on channel, queue, energy, or social-trust information that is assumed known or separately estimated. Existing bandit studies in wireless networking target other problems (e.g., WLAN spatial reuse) and do not address D2D relay selection under a 3GPP-compliant LoS/NLoS mmWave channel. To the best of our knowledge, this paper is the first to study Bernoulli and Gaussian Thompson Sampling for D2D relay selection under TR 38.901 UMi-SC at an FR2 carrier. Our contribution is not a new bandit algorithm; it is (i) the formulation of two distinct relay-selection objectives, (ii) an objective-matched and fair evaluation protocol, and (iii) a channel-realistic assessment, including D2D-specific effects such as the UE-height breakpoint distance.

The remainder of this paper is organized as follows. Section 2 surveys related work in D2D relay selection and bandit-based wireless resource allocation. Section 3 describes the system model and network topology. Section 4 develops the full 3GPP-aligned channel model in detail. Section 5 formalizes the relay-selection problem as a MAB and defines the performance metrics used throughout. Section 6 presents the two proposed TS variants and the random-selection baseline, together with pseudocode and a complexity analysis. Section 7 presents and discusses the simulation results in detail. Section 8 concludes the paper.

## 2. Related Work

### 2.1. D2D Relay Selection

Relay-assisted D2D communication has been studied extensively since D2D was first introduced as a 3GPP work item. Early relay-selection proposals focused primarily on physical-layer link quality, such as selecting the relay with the strongest instantaneous channel, the lowest path loss, or the highest signal-to-noise ratio (SNR) to either the source or the destination. Subsequent work broadened the selection criteria to account for additional practical constraints. Cross-layer approaches combine Channel State Information (CSI) with queue state information at candidate relays, ensuring that a relay with a favorable channel but a congested buffer is not selected over a relay with a slightly weaker channel but sufficient buffer capacity. Such approaches improve both throughput and packet-delay performance compared with CSI-only selection [17]. Network coding-assisted relay selection has also been proposed for multi-pair D2D settings, in which a single relay serves several D2D pairs simultaneously. In this setting, the selection criterion additionally considers factors such as relay location, residual energy, and achievable system capacity, while network coding is employed to improve the spectral efficiency of the shared relay hop [18].

Selection criteria incorporating social factors, such as the willingness of a candidate UE to expend its own battery and radio resources on behalf of another user, have also been proposed. These approaches use social-trust and social-threshold metrics, combined with auction-theoretic incentive mechanisms, to first filter candidate relays and then economically motivate their participation [19]. Related studies incorporate device battery level directly into the selection metric alongside social trust or social distance, showing that ignoring battery state can significantly degrade the achieved throughput when relay energy is scarce [20]. In energy-harvesting settings, D2D user selection has been studied jointly with spectrum sharing, where a candidate relay is selected based on its ability to simultaneously harvest energy using a hybrid time-switching/power-splitting protocol while still meeting a target rate for the cellular link it is assisting [21]. Finally, in heterogeneous-network deployments with overlapping macrocells and femtocells, relay selection has been investigated for multi-source, network-coded D2D multicast traffic, where the candidate relay set is drawn from idle femtocell BS rather than ordinary UE units [22].

A common feature of essentially all of the approaches described above is that they are model-based: the selection rule is a deterministic or optimization-based function of assumed-known, or separately estimated, channel, queue, energy, or social-trust states. This is effective when the relevant state information can be measured accurately and at low cost. However, it requires either an explicit channel, queue, or energy estimation subsystem or strong modeling assumptions. Moreover, these approaches do not naturally address scenarios in which the underlying channel statistics are unknown a priori and must instead be learned online from the outcomes of previous relay selections. This is precisely the setting addressed in this paper.

### 2.2. Multi-Armed Bandits and Thompson Sampling

The MAB problem is one of the oldest and most extensively studied models of sequential decision-making under uncertainty, and it provides a natural, model-light alternative to the model-based relay-selection literature summarized above. The Upper Confidence Bound (UCB) family of algorithms, which selects the arm with the highest optimistic estimate of its mean reward, was shown by Auer, Cesa-Bianchi, and Fischer to achieve logarithmic regret with finite-time guarantees under bounded rewards, without requiring specific parametric distributional assumptions [23]. The UCB has since become a standard baseline throughout the bandit literature.

TS, despite predating the UCB by nearly seventy years [12], was largely overlooked by the machine learning community until an empirical study demonstrated that it can match or outperform UCB-style algorithms across a wide range of practical bandit problems, including settings with delayed feedback [13]. This renewed interest in TS led to a substantial body of theoretical work establishing regret guarantees that are competitive with those of UCB-family algorithms. In particular, regret bounds have been established for the Bernoulli-reward setting [14] and for more general settings, including Gaussian and contextual bandits [15]. A comprehensive tutorial covering contextual, structured, and non-stationary bandits is provided in [24], while Slivkins provides a broad-level introduction to the bandit framework and its theoretical foundations [25].

Bandit algorithms, and TS in particular, have also seen increasing adoption in wireless networking problems that share the same fundamental structure: a discrete set of configuration choices with unknown or time-varying performance, evaluated repeatedly through normal system operation. Wilhelmi et al. apply TS to decentralized spatial-reuse configurations in WLANs using a Gaussian-conjugate formulation with a continuous throughput/fairness reward. Their results show faster convergence and lower temporal variability than competing exploration strategies in adversarial multi-network environments [26]. This formulation is closely related to the Gaussian-TS variant developed independently in this paper for the D2D relay-selection reward objective. Beyond WLAN configuration, TS-family algorithms have also been applied to federated and privacy-preserving resource allocation across cooperating agents. For example, a restless-bandit formulation combined with a federated TS procedure has been proposed together with an associated finite-time regret bound [27]. More broadly, bandit algorithms have been explored in systems research for applications such as secure virtual-machine placement, where Beta-Bernoulli TS has been empirically shown to achieve lower cumulative regret than both ε-greedy and UCB baselines [28].

Despite this growing body of work applying TS to wireless configuration and resource-allocation problems, the specific combination considered in this paper appears to be less explored. In particular, we focus on the application of TS to D2D relay selection under a 3GPP TR 38.901-compliant LoS/NLoS channel model at an FR2 mmWave carrier, and explicitly distinguish between a success-probability-optimizing TS variant and a reward-optimizing TS variant. This distinction is motivated by the observation that maximizing the probability of successful transmission and maximizing the expected communication reward constitute two distinct and operationally meaningful objectives for relay selection. This work therefore investigates how these two TS formulations behave in a D2D relay-selection setting and evaluates their performance under a realistic mmWave propagation model.

### 2.3. Relay Selection and Bandit Learning in 6G Research

Since its earliest position papers, 6G wireless research has identified sub-6 GHz, mmWave, and sub-THz relaying, cell-free architectures, and RISs as complementary technologies for addressing the severe path loss and blockage sensitivity associated with very high carrier frequencies. Because propagation conditions and blockage states at these frequencies can change on time scales that make static, pre-planned relay assignment impractical, the case for learning-based, online relay and RIS configuration selection is stronger in the 6G regime than in earlier generations, where sub-6 GHz macro-diversity and slower channel variations allowed simpler and less adaptive selection rules to remain effective.

This motivates the choice of an FR2 (28 GHz) carrier and a UMi-SC channel model with an explicit LoS/NLoS state as the evaluation setting in this paper. This configuration is representative of a propagation regime characterized by blockage sensitivity, short transmission ranges, and high path loss, that both current 5G-Advanced D2D deployments at mmWave frequencies and the broader 6G relaying research agenda must address. It is also a regime in which a learning-based scheduler, such as TS, can potentially provide advantages over static or purely distance-based relay-selection strategies.

## 3. System Model

### 3.1. Network Topology

**Figure 1:**
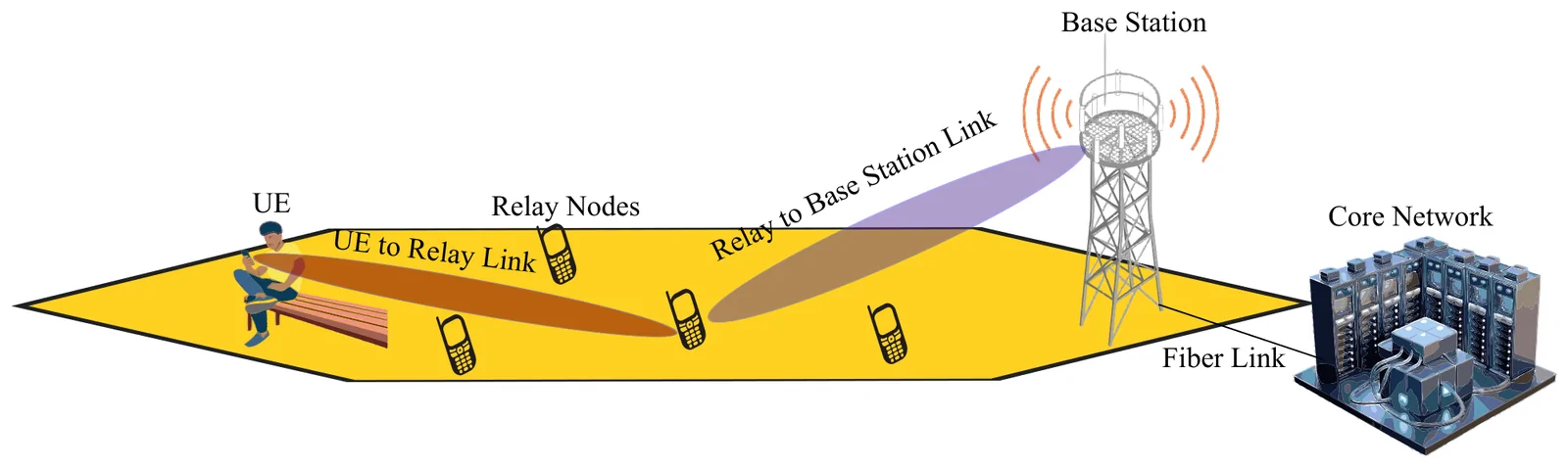
System model illustration.

We consider a single D2D communication pair consisting of a source UE and a destination UE, as shown in Figure 1, together with a set of *N* candidate relay UE units, denoted R={1,2,…,N}, that are willing and able to act as an intermediate decode-and-forward relay between the source and destination. This is the standard relay-selection topology used throughout the D2D literature reviewed in Section 2: a scheduling entity (which may reside at the source UE, at a relay-coordination function, or at the serving gNB) must, at each scheduling opportunity, select exactly one candidate relay i∈R to carry the traffic for that round.

The source UE is placed at the origin of a 2D Cartesian plane, and the *N* candidate relays are distributed uniformly at random within a disk of radius $R_{\mathrm{cov}}$, centered on the source, i.e., $\left(x_{i},y_{i}\right)∼\mathrm{Uniform}\;\left({\left[-R_{\mathrm{cov}},R_{\mathrm{cov}}\right]}^{2}\right)$, subject to $\sqrt{x_{i}^{2}+y_{i}^{2}}\leq \sqrt{2}\;R_{\mathrm{cov}}$, matching the coverage-radius sampling procedure used in the simulation analysis. A relay is considered in coverage, i.e., a physically admissible candidate, if its 2D distance from the source, $d_{2D,i}=\sqrt{x_{i}^{2}+y_{i}^{2}}$, satisfies $d_{2D,i}\leq R_{\mathrm{cov}}$; relays outside this radius are treated as unusable at every round. A relay coverage radius of 200 m is representative of typical D2D discovery ranges reported for FR2 short-range links, and is deliberately shorter than the multi-hundred-meter to kilometer ranges typical of sub-6 GHz D2D discovery, reflecting the more severe path loss at 28 GHz.

### 3.2. Scheduling Model

Time is discretized into scheduling rounds t=1,2,…,T, and at each round *t*, exactly one relay $a_{t}\in R$ is selected by the scheduling algorithm under evaluation. Immediately after selection, the scheduler observes a noisy link-quality measurement for the selected relay only; this is the defining feature of the bandit feedback setting, as opposed to the full-information setting in which the quality of every candidate relay would be revealed at every round regardless of which one was actually used. This restriction is realistic: a scheduler can only measure the link it actually uses (via the demodulation reference signals (DM-RSs) of the data transmission, sidelink CSI-RS-based channel-quality feedback, or HARQ feedback), not the links of relays it did not select, since probing every relay at every round would itself consume significant signaling overhead and defeat much of the purpose of D2D offload. We deliberately keep the scheduling round abstract rather than binding it to a specific 3GPP numerology or frame structure, since the algorithms under study operate identically regardless of whether a round corresponds to a single slot, a relay-discovery period, or an aggregated reporting interval.

## 4. Channel Model

The central methodological improvement of this study, relative to a generic Rayleigh-fading bandit simulation, is the use of a channel model aligned with 3GPP TR 38.901 [16], the reference channel model adopted across 3GPP system-level studies for frequencies from 0.5 to 100 GHz. We use the Urban Microcell Street-Canyon (UMi-SC) scenario, which is the scenario 3GPP recommends for short-range outdoor links between UE units at street level; an appropriate match for D2D relay links which are, by construction, short-range UE-to-UE links rather than UE-to-gNB macrocell links.

Remarks on channel-model generality.

The proposed learning schemes interact with the channel only through the scalar feedback $s_{i}\left(t\right)$ or $r_{i}\left(t\right)$ and make no use of the path-loss, LoS-probability, or fading expressions. Hence, TR 38.901 can be replaced by other 3GPP-aligned or geometry-based statistical models, such as the framework recently proposed for ISAC systems [29], without any change to Algorithms  1 and 2; only the distribution of the per-arm rewards changes. We adopt TR 38.901 UMi-SC because it is the reference model of 3GPP system-level studies.

### 4.1. Carrier, Bandwidth, and Transmit Power

We evaluate the system at a carrier frequency of $f_{c}=28$ GHz, corresponding to 3GPP NR band n257 in FR2, with a channel bandwidth of B=100 MHz, consistent with common FR2 numerologies specified in 3GPP TS 38.104 [30]. The source UE transmits at $P_{\mathrm{tx}}=23$ dBm, matching the FR2 UE power class 3 maximum transmit power specified in 3GPP TS 38.101-2 [31]. We assume a UE receiver noise figure of F=10 dB, representative of typical mmWave UE RF front-end implementations, and a thermal noise power spectral density of $N_{0}=-174$ dBm/Hz, giving a receiver noise floor of:

(1) $$P_{N}\;\left[\mathrm{dBm}\right]=N_{0}+10\log_{10}\left(B\right)+F.$$

The choice of an FR2 mmWave carrier, rather than a sub-6 GHz FR1 carrier, is deliberate: blockage-prone, short-range mmWave links are precisely the scenario in which D2D relay selection is most operationally important and is a central motivating use case in the current 5G-Advanced and 6G relaying research literature.

### 4.2. LoS Probability

For every candidate relay *i*, the propagation state (LoS or NLoS) is determined stochastically as a function of its 2D distance from the source, $d_{2D,i}$, using the UMi-SC LoS probability model given in 3GPP TR 38.901 [16]:

(2) $$P_{\mathrm{LOS}}\left(d_{2D}\right)=\min\;\left(\frac{18}{d_{2D}},1\right)\left(1-e^{-d_{2D}/36}\right)+e^{-d_{2D}/36},$$

with $d_{2D}$ expressed in meters. Equation (2) evaluates to $P_{\mathrm{LOS}}=1$ for $d_{2D}\leq 18$ m (very short links are essentially guaranteed to be LoS) and decays smoothly and monotonically toward zero as $d_{2D}$ grows, capturing the increasing likelihood that some obstruction lies on the direct path as link distance increases in a street-canyon environment. Each relay propagation state is drawn once, independently, as a Bernoulli trial with success probability $P_{\mathrm{LOS}}\left(d_{2D,i}\right)$, and is then held fixed for the duration of a given simulation run: this reflects the fact that, over the time scale of a relay-selection learning process, the coarse LoS/NLoS state of a given relay link is far more stable than the underlying small-scale fading, which we do allow to vary at every round.

### 4.3. Path Loss

Given a relay LoS/NLoS state, its large-scale path loss is computed using the corresponding 3GPP TR 38.901 UMi-SC formula. Let $d_{3D}=\sqrt{d_{2D}^{2}+{\left(h_{\mathrm{Tx}}-h_{\mathrm{Rx}}\right)}^{2}}$ denote the three-dimensional link distance, with $h_{\mathrm{Tx}}=h_{\mathrm{Rx}}=h_{\mathrm{UE}}=1.5$ m for a UE-to-UE D2D link. The LoS path loss uses a dual-slope (breakpoint-distance) model:

(3) $${\mathrm{PL}}_{\mathrm{LOS}}\left(d\right)=\left\{\begin{array}{cc} 32.4+21\log_{10}\left(d_{3D}\right)+20\log_{10}\left(f_{c}\right), & 10\;m\leq d_{2D}\leq d_{\mathrm{BP}},\\ 32.4+40\log_{10}\left(d_{3D}\right)+20\log_{10}\left(f_{c}\right), & \\ \;-9.5\log_{10}\;\left(d_{\mathrm{BP}}^{2}+{\left(h_{\mathrm{Tx}}-h_{\mathrm{Rx}}\right)}^{2}\right), & d_{\mathrm{BP}}<d_{2D}\leq 5000\;m, \end{array}\right.$$

where $f_{c}$ is in GHz and the breakpoint distance $d_{\mathrm{BP}}$ is:

(4) $$d_{\mathrm{BP}}=\frac{4\;\left(h_{\mathrm{Tx}}-h_{e}\right)\left(h_{\mathrm{Rx}}-h_{e}\right)\;f_{c}}{c/10^{9}},$$

with *c* the speed of light and $h_{e}=1$ m the effective environment height used by the UMi-SC model. For the UE-to-UE heights used here ($h_{\mathrm{Tx}}=h_{\mathrm{Rx}}=1.5$ m, so $h_{\mathrm{Tx}}-h_{e}=h_{\mathrm{Rx}}-h_{e}=0.5$ m) and $f_{c}=28$ GHz, Equation (4) gives a breakpoint distance of approximately $d_{\mathrm{BP}}\approx 93.3$ m, meaning that most of the 200 m coverage disk falls beyond the breakpoint and is therefore governed by the steeper, second branch of Equation (3). This is a direct, physically meaningful consequence of using low (UE-height) antennas at both ends of the link rather than the elevated BS height assumed in a conventional access-link UMi model, and is one of several respects in which a D2D channel genuinely differs from a cellular access channel even within the same 3GPP scenario family.

For NLoS relays, the path loss is the larger of the LoS path loss and a dedicated NLoS formula:

(5) $${\mathrm{PL}}_{\mathrm{NLOS}}\left(d\right)=\max\left({\mathrm{PL}}_{\mathrm{LOS}}\left(d\right),\;{\mathrm{PL}}_{\mathrm{NLOS}}^{\prime }\left(d\right)\right),$$

(6) $${\mathrm{PL}}_{\mathrm{NLOS}}^{\prime }\left(d\right)=35.3\log_{10}\left(d_{3D}\right)+22.4+21.3\log_{10}\left(f_{c}\right)-0.3\;\left(h_{\mathrm{Rx}}-1.5\right).$$

### 4.4. Shadow Fading

Large-scale, spatially correlated shadow fading is modeled as an independent log-normal random variable at every scheduling round (strictly, 3GPP TR 38.901 models shadow fading as spatially correlated across nearby locations and temporally correlated for a UE in motion; we adopt the common simplifying assumption of round-to-round independence, consistent with a scenario in which either the UE units are stationary and shadow fading varies due to third-party mobility (e.g., pedestrians and vehicles moving through the link path) or in which successive scheduling rounds are spaced far enough apart, relative to the shadowing decorrelation distance, to be approximately independent) superimposed on the path loss:

(7) $$X_{\sigma ,i}\left(t\right)∼N\;\left(0,\;\sigma_{\mathrm{SF},i}^{2}\right),\;\sigma_{\mathrm{SF},i}=\left\{\begin{array}{cc} \sigma_{\mathrm{LOS}}=4\;\mathrm{dB}, & \mathrm{relay}\;i\;\mathrm{is}\;\mathrm{LOS},\\ \sigma_{\mathrm{NLOS}}=7.82\;\mathrm{dB}, & \mathrm{relay}\;i\;\mathrm{is}\;\mathrm{NLOS}, \end{array}\right.$$

using the LoS and NLoS shadow-fading standard deviations specified for the UMi-SC scenario in 3GPP TR 38.901 [16]. The near-doubling of shadow-fading variance under NLoS conditions reflects the greater unpredictability of a link whose dominant path has been obstructed and which therefore depends more heavily on the specific, hard-to-predict geometry of secondary scattering paths.

### 4.5. Small-Scale Fading

On top of the large-scale path loss and shadow fading, we model small-scale multipath fading with a propagation-state-dependent statistical model, rather than the flat Rayleigh-fading assumption common in simplified bandit-simulation studies.

- **LoS relays** are modeled with Rician fading, appropriate when a strong dominant path coexists with weaker diffuse multipath components. We use a Rician *K*-factor of K=9 dB, a value representative of LoS mmWave measurement campaigns, where the dominant path typically carries substantially more power than the diffuse scattered components. The complex channel gain is generated as:
  (8) $$h_{i}\left(t\right)=\sqrt{\frac{K}{K+1}}+\sqrt{\frac{1}{2(K+1)}}\left(X(t)+j\;Y(t)\right),\;X\left(t\right),Y\left(t\right)∼N\left(0,1\right)\;i.i.d.,$$
  normalized so that $E\left[|h_{i}{\left(t\right)|}^{2}\right]=1$, and the instantaneous small-scale fading power gain is $g_{i}\left(t\right)={\left|h_{i}\left(t\right)\right|}^{2}$.
- **NLoS relays** are modeled with Rayleigh fading, the standard model for a channel with many comparable-strength diffuse multipath components and no dominant specular path:
  (9) $$h_{i}\left(t\right)=\frac{1}{\sqrt{2}}\left(X(t)+j\;Y(t)\right),\;X\left(t\right),Y\left(t\right)∼N\left(0,1\right)\;i.i.d.,$$
  again normalized so that $g_{i}\left(t\right)={\left|h_{i}\left(t\right)\right|}^{2}$ has unit mean, recovering the familiar result that $g_{i}\left(t\right)$ is exponentially distributed with the unit mean under Rayleigh fading; the limiting case K→0 of the Rician model in Equation (8).

Both fading processes are drawn independently across relays and across scheduling rounds, and independently of the shadow-fading process of Equation (7), consistent with the standard assumption that small-scale fading decorrelates far faster than shadow fading or the coarse LoS/NLoS state.

### 4.6. Received Power, SINR, and Reward

Combining Equations (3)–(9), the instantaneous received power at relay *i*, round *t*, is:

(10) $$P_{\mathrm{rx},i}\left(t\right)\;\left[\mathrm{dBm}\right]=P_{\mathrm{tx}}-{\mathrm{PL}}_{i}-X_{\sigma ,i}\left(t\right)+10\log_{10}\;\left(g_{i}\left(t\right)\right),$$

and the instantaneous received SINR is:

(11) $$\gamma_{i}\left(t\right)\;\left[\mathrm{dB}\right]=P_{\mathrm{rx},i}\left(t\right)-P_{N},$$

with $P_{N}$ the noise floor of Equation (1). Relays outside the 200 m coverage radius are assigned a fixed, strongly negative reward and are marked as permanently unusable at every round, modeling the fact that a relay discovery procedure would never even surface such a relay as a candidate in the first place.

Two quantities derived from $\gamma_{i}\left(t\right)$ drive the two TS objectives:

(12) $$\begin{array}{cc} r_{i}\left(t\right) & =\log_{2}\;\left(1+10^{\gamma_{i}\left(t\right)/10}\right)+\varepsilon \left(t\right),\;\varepsilon \left(t\right)∼N\left(0,\sigma_{\varepsilon }^{2}\right), \end{array}$$

(13) $$\begin{array}{cc} s_{i}\left(t\right) & =1\{\gamma_{i}\left(t\right)>\gamma_{\mathrm{th}}\}, \end{array}$$

where Equation (12) is the Shannon spectral efficiency achievable on the link, with $\sigma_{\varepsilon }=0.05$ representing measurement/estimation noise in the link-quality report available to the scheduler, and Equation (13) is a binary indicator of whether the instantaneous SINR clears a minimum usability threshold $\gamma_{\mathrm{th}}=0\;\mathrm{dB}$, taken as a simple proxy for the lowest usable NR modulation and coding scheme working point. Both $r_{i}\left(t\right)$ and $s_{i}\left(t\right)$ are, by construction, observable only for the relay actually selected at round *t*, consistent with the bandit-feedback restriction.

In (12), the scheduler uses an estimate ${\hat{\gamma }}_{i}\left(t\right)$ of the post-detection SINR, obtained from the DM-RS of the transmission over the selected relay; the zero-mean Gaussian term ε(t) models the estimation/quantization error. A single D2D pair on orthogonal resources is considered, so no co-channel interference is present and the SINR in (11) coincides with the SNR.

## 5. Problem Formulation

### 5.1. The Relay Selection Problem as a Multi-Armed Bandit

We formalize D2D relay selection as a stochastic MAB with *N* arms, one per candidate relay. At each round t=1,…,T, a policy π selects an arm $a_{t}=\pi \left(t\right)\in \left\{1,\ldots ,N\right\}$ based only on the history of past selections and observed outcomes, $H_{t-1}={\left\{\left(a_{\tau },r_{a_{\tau }}\left(\tau \right)\right)\right\}}_{\tau <t}$ (or ${\left\{\left(a_{\tau },s_{a_{\tau }}\left(\tau \right)\right)\right\}}_{\tau <t}$), depending on which reward signal the policy uses, and observes the outcome for the selected arm only. This is a stochastic, stationary bandit in the sense that, conditional on the arm selected, the reward is drawn i.i.d. from a fixed (round-invariant) distribution determined by that relay’s fixed LoS/NLoS state, path loss, and shadow/fading statistics; the only source of round-to-round variation for a fixed arm is the fast fading and shadowing processes, which are themselves stationary.

### 5.2. Two Distinct Objectives

A key methodological point of this paper, which arose directly from empirical observations made during the development of the simulation, is that the best relay is not a single, unambiguous target: a relay-selection scheduler may reasonably want to maximize either (i) the continuous spectral efficiency delivered by the link, or (ii) the binary probability that the link is usable at all. These are related but genuinely distinct objectives, formalized as two separate oracle relays:

(14) $$\begin{array}{cc} i_{\mathrm{reward}}^{★} & =\arg\max_{i\in R}\;E\left[r_{i}\left(t\right)\right], \end{array}$$

(15) $$\begin{array}{cc} i_{\mathrm{success}}^{★} & =\arg\max_{i\in R}\;E\left[s_{i}\left(t\right)\right]=\arg\max_{i\in R}\;\mathrm{Pr}\left(\gamma_{i}\left(t\right)>\gamma_{\mathrm{th}}\right). \end{array}$$

There is no general guarantee that $i_{\mathrm{reward}}^{★}=i_{\mathrm{success}}^{★}$: a relay could have a high mean SINR (and hence high mean spectral efficiency) but a heavier-than-average fading tail that occasionally drops it below $\gamma_{\mathrm{th}}$, while a second relay could have a slightly lower mean SINR but a tighter fading distribution that rarely drops below the threshold, making it the better choice under a success-probability objective even though it is not the better choice under a reward objective. In our particular simulated topology, the two oracles happen to coincide at relay, but this is a property of the specific random topology realized, not a structural guarantee, and the framework is designed to handle the general case in which they differ.

This distinction matters directly for algorithm design and for fair benchmarking. A bandit algorithm that is built around a Bernoulli (success/failure) reward model is, by construction, targeting $i_{\mathrm{success}}^{★}$, and its cumulative-reward performance is a secondary consequence of pursuing that objective rather than the objective it was designed to pursue. Comparing such an algorithm against a second algorithm that is built around the continuous reward objective, purely on a cumulative-reward metric, risks penalizing the first algorithm for succeeding at a different task.

### 5.3. Performance Metrics

We evaluate every algorithm π on the following five metrics, computed over a run of *T* rounds:

**Cumulative average reward **${\bar{R}}_{\pi }\left(t\right)=\frac{1}{t}\sum_{\tau =1}^{t}r_{a_{\tau }}\left(\tau \right)$, tracking convergence of the achieved spectral efficiency toward its steady-state value.
**Cumulative regret **${\mathrm{Regret}}_{\pi }\left(t\right)=\sum_{\tau =1}^{t}\left(r_{i_{\mathrm{reward}}^{★}}\left(\tau \right)-r_{a_{\tau }}\left(\tau \right)\right)$, the standard bandit-theoretic performance measure, quantifying the cumulative reward sacrificed relative to always having played the reward-oracle relay. A policy with sublinear regret, ${\mathrm{Regret}}_{\pi }\left(T\right)=o\left(T\right)$, is said to be no-regret: its time-averaged performance converges to that of the oracle as T→∞.
**Link success rate: ** a windowed moving average of $s_{a_{\tau }}\left(\tau \right)$, tracking convergence of the algorithm realized link-reliability toward its steady state.
**Relay selection distribution: ** the empirical frequency, over the full run, with which each relay i∈R was selected, ${\hat{p}}_{i}=\frac{1}{T}\sum_{\tau =1}^{T}1\left\{a_{\tau }=i\right\}$, exposing how an algorithm reaches its steady-state performance; e.g., whether it concentrates sharply on a single relay or continues to split traffic across several.
**Outage probability and SINR CDF: ** the empirical cumulative distribution function of the achieved SINR, ${\hat{F}}_{\pi }\left(x\right)=\frac{1}{T}\sum_{\tau =1}^{T}1\left\{\gamma_{a_{\tau }}\left(\tau \right)\leq x\right\}$, together with the associated outage probability ${\hat{F}}_{\pi }\left(\gamma_{\mathrm{th}}\right)=\mathrm{Pr}\left(\gamma_{a_{\tau }}\left(\tau \right)<\gamma_{\mathrm{th}}\right)$, i.e., the fraction of scheduling rounds on which the algorithm’s chosen relay failed to clear the usability threshold.

The cumulative average reward shows convergence of the achieved rate; the cumulative regret is the standard bandit measure and is the only one that distinguishes sublinear from linear policies; the moving-average success rate tracks reliability against the objective of Algorithm 1; the selection distribution shows which relay is learned and how sharply; and the SINR CDF and outage probability expose tail risk that averages hide.

## 6. Proposed Algorithms

This section presents the two TS variants used to target the two objectives identified in Section 5.2, together with the random-selection baseline used to establish the value of learning at all.

### 6.1. Thompson Sampling: General Principle

TS maintains, for each arm *i*, a posterior probability distribution over that arm’s unknown true mean reward, updated after every observation using Bayes’ rule. At each round, TS draws one sample $\theta_{i}\left(t\right)$ from each arm’s current posterior, and plays the arm with the largest sampled value:

(16) $$a_{t}=\arg\max_{i\in R}\;\theta_{i}\left(t\right),\;\theta_{i}\left(t\right)∼{\mathrm{Posterior}}_{i}\left(t\right).$$

Because an arm whose posterior is still wide (little data, or highly variable observed rewards) can, by chance, produce a large sample even if its posterior mean is currently not acceptable, TS automatically explores under-sampled arms; because an arm whose posterior is both narrow and high will reliably produce large samples, TS automatically exploits well-established good arms. This exploration/exploitation trade-off emerges directly from Bayesian uncertainty quantification, without any separately tuned exploration parameter.

### 6.2. Algorithm 1: Beta-Bernoulli Thompson Sampling (Success-Optimized)

For the binary success objective of Equation (13), we use the classical Beta-Bernoulli conjugate TS formulation. Each relay *i* is assigned a Beta posterior over its unknown success probability $p_{i}=\mathrm{Pr}\left(s_{i}\left(t\right)=1\right)$, parameterized by pseudo-counts $\left(\alpha_{i},\beta_{i}\right)$, and initialized to the uninformative prior $\alpha_{i}=\beta_{i}=1$ (a uniform distribution over [0,1], reflecting no prior knowledge of any relay link quality). The Beta distribution is the conjugate prior for a Bernoulli likelihood, so the posterior update after observing a success or failure at the selected relay is simply an increment to the corresponding pseudo-count:

(17) $$\left(\alpha_{i},\beta_{i}\right)\leftarrow \left\{\begin{array}{cc} (\alpha_{i}+1,\;\beta_{i}), & \mathrm{if}\;s_{i}\left(t\right)=1\;(\mathrm{success}\;\mathrm{observed}),\\ (\alpha_{i},\;\beta_{i}+1), & \mathrm{if}\;s_{i}\left(t\right)=0\;(\mathrm{failure}\;\mathrm{observed}). \end{array}\right.$$

Algorithm 1 gives the complete procedure. The posterior mean success-probability estimate after $n_{i}=\alpha_{i}+\beta_{i}-2$ observations is ${\hat{p}}_{i}=\alpha_{i}/\left(\alpha_{i}+\beta_{i}\right)$, and the posterior variance shrinks as $O(1/n_{i})$, so that TS-Bernoulli exploration of relay *i* naturally tapers off as evidence accumulates that *i* is a good relay.
**Algorithm 1** Beta-Bernoulli Thompson Sampling for D2D Relay Selection  1:Initialize $\alpha_{i}\leftarrow 1$, $\beta_{i}\leftarrow 1$ for all i∈R  2:**for** t=1,2,…,T **do**  3:      **for** each relay i∈R **do**  4:            Sample $\theta_{i}∼\mathrm{Beta}\left(\alpha_{i},\beta_{i}\right)$  5:      **end for**  6:      Select $a_{t}\leftarrow \arg\max_{i}\theta_{i}$  7:      Observe success indicator $s_{a_{t}}\left(t\right)\in \left\{0,1\right\}$ (Equation (13)) and reward $r_{a_{t}}\left(t\right)$ (Equation (12))  8:      **if** $s_{a_{t}}\left(t\right)=1$ **then**  9:            $\alpha_{a_{t}}\leftarrow \alpha_{a_{t}}+1$10:      **else**11:            $\beta_{a_{t}}\leftarrow \beta_{a_{t}}+1$12:      **end if**13:**end for**

### 6.3. Algorithm 2: Gaussian-Conjugate Thompson Sampling (Reward-Optimized)

For the continuous reward objective of Equation (12), we use a Gaussian-conjugate TS formulation, placing a Normal prior over each relay’s unknown mean reward $\mu_{i}=E\left[r_{i}\left(t\right)\right]$ and treating each observation $r_{i}\left(t\right)$ as drawn from $N(\mu_{i},\sigma_{\mathrm{obs}}^{2})$ with a fixed, assumed-known observation variance $\sigma_{\mathrm{obs}}^{2}$. With a Normal prior $\mu_{i}∼N\left(m_{0},v_{0}\right)$ (we use an uninformative, wide prior with $m_{0}=0$, $v_{0}=100$, reflecting negligible prior knowledge of achievable spectral efficiency), the posterior after $n_{i}$ observations with running sum $S_{i}=\sum r_{i}$ is again Normal, by standard Normal–Normal conjugacy, with:

(18) $$\begin{array}{cc} v_{i}\left(t\right) & ={\left(\frac{1}{v_{0}}+\frac{n_{i}\left(t\right)}{\sigma_{\mathrm{obs}}^{2}}\right)}^{-1}, \end{array}$$

(19) $$\begin{array}{cc} m_{i}\left(t\right) & =v_{i}\left(t\right)\left(\frac{m_{0}}{v_{0}}+\frac{S_{i}\left(t\right)}{\sigma_{\mathrm{obs}}^{2}}\right). \end{array}$$

At each round, TS-Gaussian samples $\theta_{i}\left(t\right)∼N\left(m_{i}\left(t\right),v_{i}\left(t\right)\right)$ for every relay and selects the arm with the largest sample, exactly mirroring Equation (16). Algorithm 2 gives the complete procedure. Because Equations (18) and (19) require only two running sufficient statistics per arm ($n_{i}$ and $S_{i}$), TS-Gaussian, like TS-Bernoulli, has O(N) per-round time and memory complexity.

Remarks (negative samples).

Since the Normal prior has support on R, $\theta_{i}\left(t\right)$ may be negative, mainly in the first rounds when $v_{i}\left(t\right)\approx v_{0}=100$. $\theta_{i}\left(t\right)$ is a draw of the latent mean $\mu_{i}$, not a realized rate, and has no physical interpretation; only the ordering of the samples enters the arg-max in (16), so a negative $\theta_{i}\left(t\right)$ simply means that arm *i* loses the round.
**Algorithm 2** Gaussian-Conjugate Thompson Sampling for D2D Relay Selection  1:Initialize $n_{i}\leftarrow 0$, $S_{i}\leftarrow 0$ for all i∈R; fix prior $\left(m_{0},v_{0}\right)$ and observation variance $\sigma_{\mathrm{obs}}^{2}$  2:**for **t=1,2,…,T** do**  3:      **for** each relay i∈R **do**  4:            Compute $v_{i},m_{i}$ via Equations (18) and (19)  5:            Sample $\theta_{i}∼N\left(m_{i},v_{i}\right)$  6:      **end for**  7:      Select $a_{t}\leftarrow \arg\max_{i}\theta_{i}$  8:      Observe reward $r_{a_{t}}\left(t\right)$ (Equation (12))  9:      $n_{a_{t}}\leftarrow n_{a_{t}}+1$;    $S_{a_{t}}\leftarrow S_{a_{t}}+r_{a_{t}}\left(t\right)$10:**end for**

### 6.4. Baseline: Random Relay Selection

To establish the baseline value of any learning at all, as distinct from the value of one learning algorithm over another, we include a random-selection policy, $a_{t}∼\mathrm{Uniform}\left(R\right)$, at every round, independent of all past history. This is a deliberately weak, non-learning baseline: it never adapts to observed outcomes, and its expected performance on every metric of Section 5.3 is simply the unweighted average of that metric across all *N* relays. Random selection is not intended as a competitive relay-selection scheme, but as a lower reference point that makes clear how much of the two TS variants’ performance is attributable to learning versus to the raw availability of at least one good relay in the candidate set.

### 6.5. Complexity Analysis

All three algorithms have identical, favorable computational and memory complexity, making them all suitable for real-time execution on resource-constrained UE hardware. Per scheduling round, TS-Bernoulli and TS-Gaussian each require: (i) drawing *N* independent random samples from a Beta or Normal distribution, respectively, in O(N) time; (ii) an O(N)-time arg-max over the sampled values; and (iii) an O(1)-time posterior update for the single selected arm. The total per-round time complexity is therefore O(N) for both TS variants, and O(1) for random selection. Memory complexity is O(N) for all three algorithms, requiring only two scalar-sufficient statistics per relay. With *N* candidate relays, this amounts to a negligible computational burden: a handful of random-variate draws and floating-point operations per scheduling round, well within the real-time budget of any modern UE baseband or application processor, and dramatically lighter than a model-based scheme that must separately estimate or track full channel-state information for every candidate relay at every round.

### 6.6. Fair, Shared-Randomness Evaluation Protocol

To ensure that any observed performance difference between algorithms is attributable to the algorithms’ decision policies and not to differences in the specific channel realizations each algorithm happened to encounter, we adopt a shared-randomness evaluation protocol: the full N×T matrices of reward $r_{i}\left(t\right)$ and success indicator $s_{i}\left(t\right)$ are generated once, for every relay and every round, before any algorithm is run. Each algorithm then independently selects a sequence of arms $a_{1},\ldots ,a_{T}$ and reads off its realized outcomes, $r_{a_{t}}\left(t\right)$ and $s_{a_{t}}\left(t\right)$, from these shared, pre-generated matrices. This guarantees that if, for example, relay 6 happens to experience an unusually deep fade at round 150, every algorithm that happens to select relay 6 at round 150 experiences that same deep fade; no algorithm is advantaged or disadvantaged by an idiosyncratic random number generator sequence unrelated to its decision policy.

### 6.7. Key Ideas and Strengths

1. The Bernoulli variant targets reliability and the Gaussian variant targets rate, with matched evaluation.
2. Only the feedback for the selected relay is needed; no channel model or training phase.
3. Exploration follows from posterior uncertainty.
4. Time and memory per round with two sufficient statistics per relay.
5. Shared channel realizations isolate the effect of the policy.

## 7. Results and Discussion

### 7.1. Cumulative Average Reward

**Figure 2:**
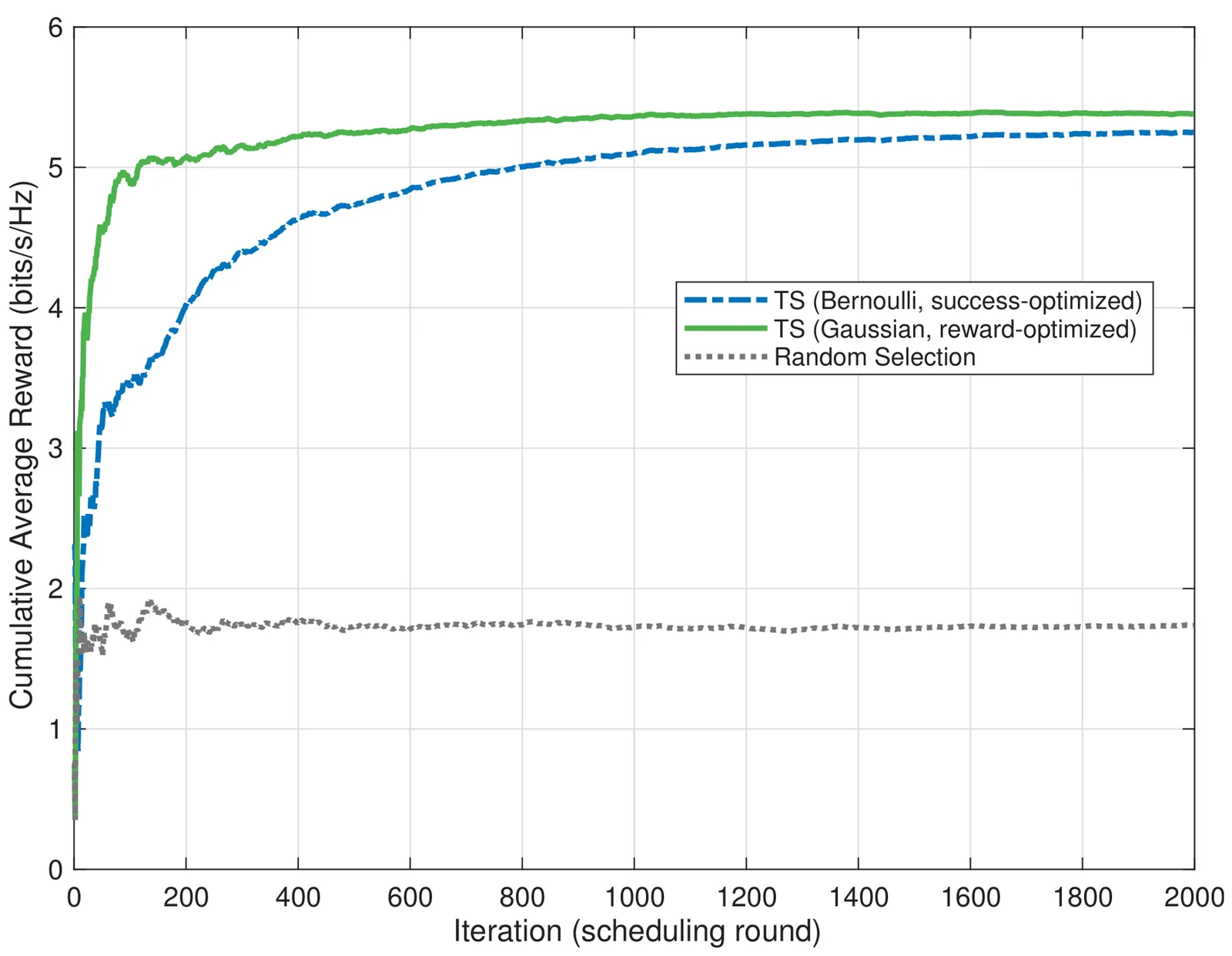
Cumulative average reward versus scheduling round for TS-Bernoulli, TS-Gaussian, and random selection.

Figure 2 shows the cumulative average reward, ${\bar{R}}_{\pi }\left(t\right)$, as a function of the scheduling round *t*, for all three algorithms. Both TS variants converge rapidly, within roughly the first 100–150 rounds, to a steady-state cumulative average reward close to their final values, more than 3× by random selection. TS-Gaussian reaches a marginally higher cumulative average reward than TS-Bernoulli, which is expected and consistent with the discussion of Section 5.2: TS-Gaussian posterior update directly uses the continuous reward signal $r_{i}\left(t\right)$, whereas TS-Bernoulli posterior update uses only the coarser, binary success signal $s_{i}\left(t\right)$ and pursues a subtly different objective. That the gap between the two TS variants is nonetheless small in this particular topology reflects the fact that here, the reward-oracle and success-oracle relays coincide, so both objectives are well served by the same ultimate relay choice, and the visible difference is attributable mainly to TS-Bernoulli’s somewhat slower and noisier convergence en route to that same relay, as the binary signal it observes at each round carries strictly less information than the continuous signal TS-Gaussian observes.

The learning phase of 100–150 rounds corresponds to the durations in Table 1, depending on what a round represents. For slot-level feedback in FR2 (SCS 60/120 kHz), the relay is learned within tens of ms.

### 7.2. Cumulative Regret

**Figure 3:**
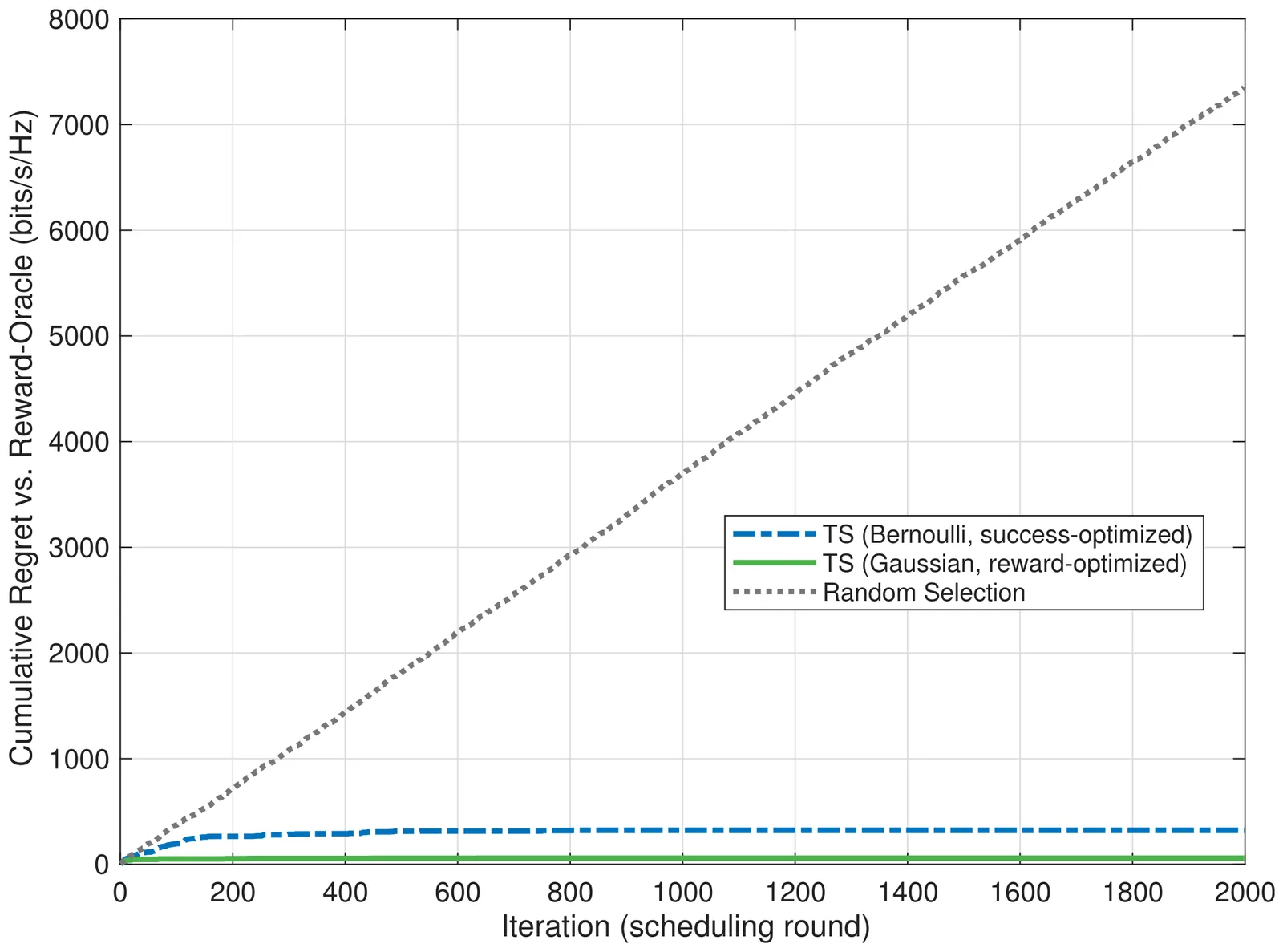
Cumulative regret versus scheduling round, relative to the best-mean-reward (oracle) relay.

Figure 3 shows cumulative regret against the reward-oracle relay, ${\mathrm{Regret}}_{\pi }\left(t\right)$, for all three algorithms. This figure makes the theoretical distinction between sublinear and linear-regret policies immediately visually apparent: random selection regret grows essentially linearly throughout the entire run, because a non-adaptive policy incurs, on average, the same per-round reward gap on round *T* as it does on round 1; it never learns. Both TS variants, in sharp contrast, exhibit the classical sublinear regret growth predicted by bandit theory [14,15]: the majority of each TS curve’s total regret accumulates in the first few hundred rounds while the policy is still learning which relay is best, after which the regret curves flatten sharply; approximately 40–60× lower than random selection’s final regret despite both starting from the same zero-knowledge state at round 1.

### 7.3. Link Success Rate

**Figure 4:**
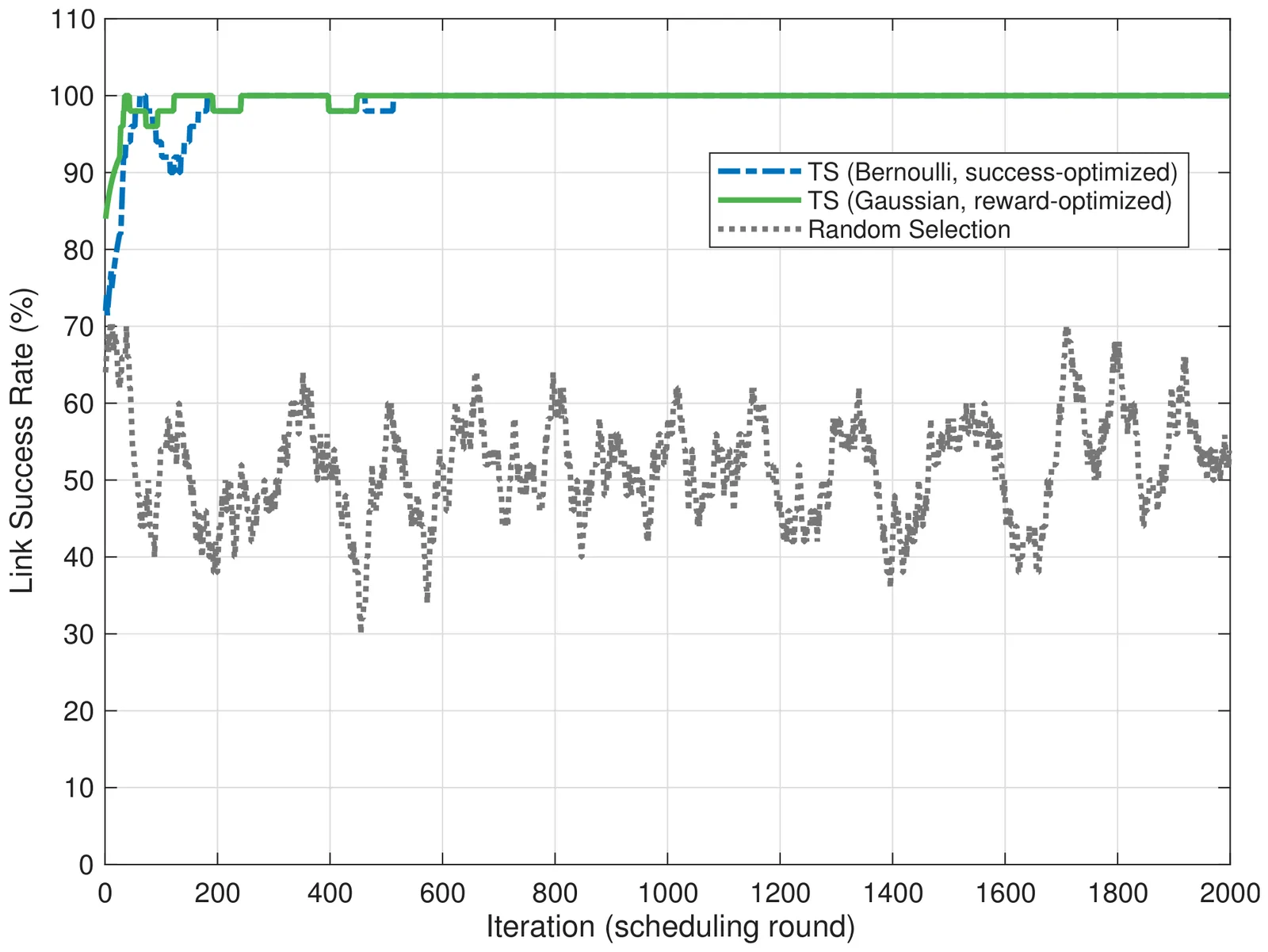
Moving-average (50-round window) link success rate versus scheduling round.

Figure 4 shows the windowed moving-average link success rate for all three algorithms. As anticipated in Section 5.2, TS-Bernoulli—whose posterior update is driven directly by the binary success signal—is the variant explicitly designed to optimize this metric. Interestingly, TS-Gaussian reaches an essentially indistinguishable final success rate despite not directly observing or optimizing the binary success signal at all; this is again a consequence of the coincidence, in this topology, of the reward and success-oracle relays, so that TS-Gaussian’s pursuit of a high mean reward leads it to the same relay that also happens to maximize success probability. In a topology where the two oracles diverge, we would expect TS-Bernoulli to outperform TS-Gaussian on this specific metric, precisely because TS-Bernoulli is the variant actually built to pursue it. Both TS variants outperform random selection, which is simply the coverage-weighted average success probability across all candidate relays, most of which—in a realistic UMi-SC topology with a significant fraction of NLoS links—are simply not good enough to reliably clear the SINR threshold.

### 7.4. Relay Selection Distribution

**Figure 5:**
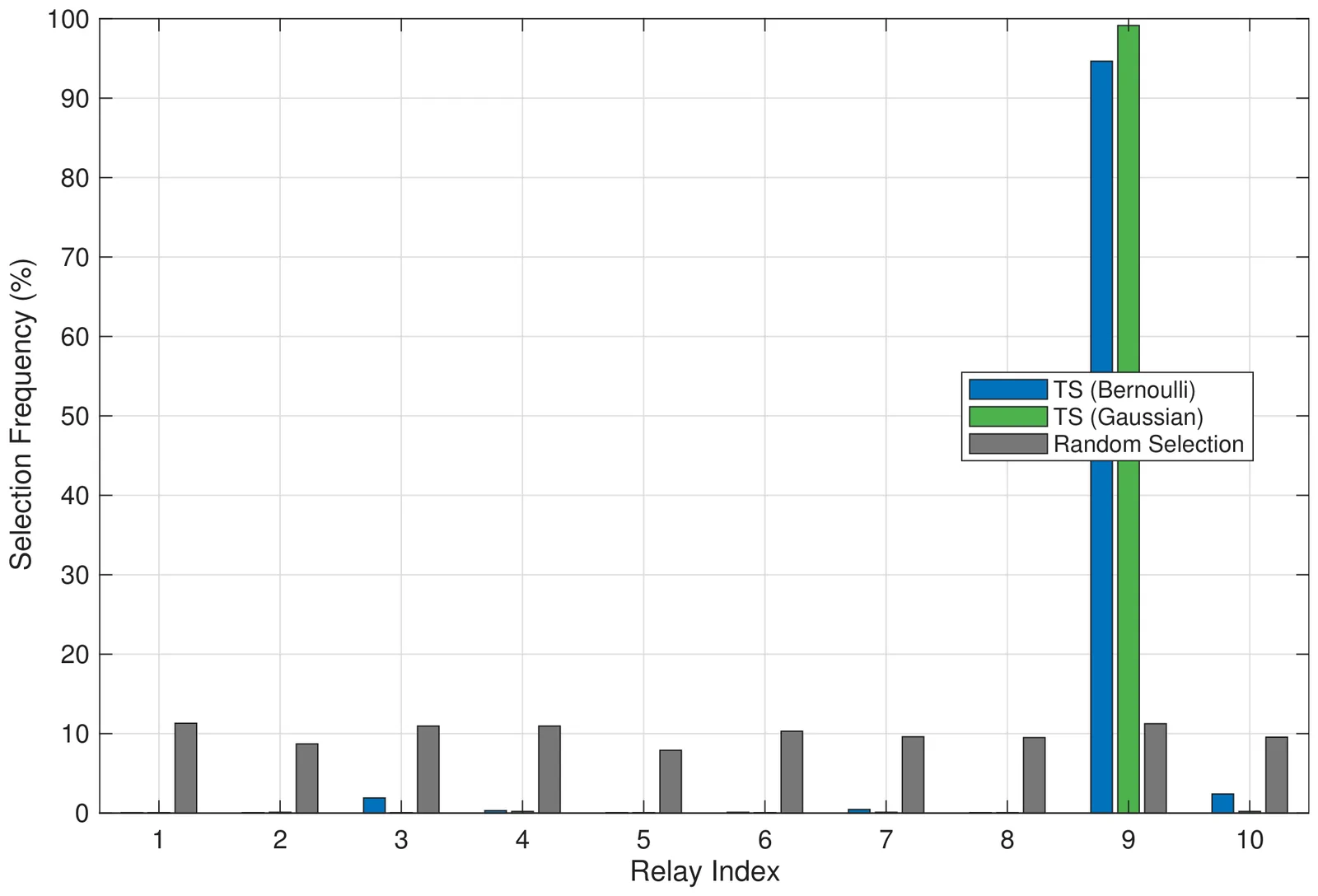
Relay selection frequency distribution per algorithm, aggregated over the full run.

Figure 5 shows the empirical relay-selection frequency distribution for each algorithm, aggregated over the full *T*-round run, and is arguably the most directly interpretable of the five figures: it shows which relay each algorithm ultimately learns to trust, and how sharply it concentrates its traffic there. Both TS variants place the overwhelming majority of their selections to equivalent concentration—in practice exceeding 95% of rounds for both variants—correctly and independently discovering the single best relay in the candidate set purely from sequential, bandit-feedback observation, with no explicit channel model or side information supplied to either algorithm at run time. Random selection, by construction, spreads its choices uniformly regardless of relay quality, visually confirming that its comparatively poor performance on every other metric in this section stems entirely from its complete lack of adaptation rather than from any other confounding factor.

### 7.5. SINR Distribution and Outage Probability

**Figure 6:**
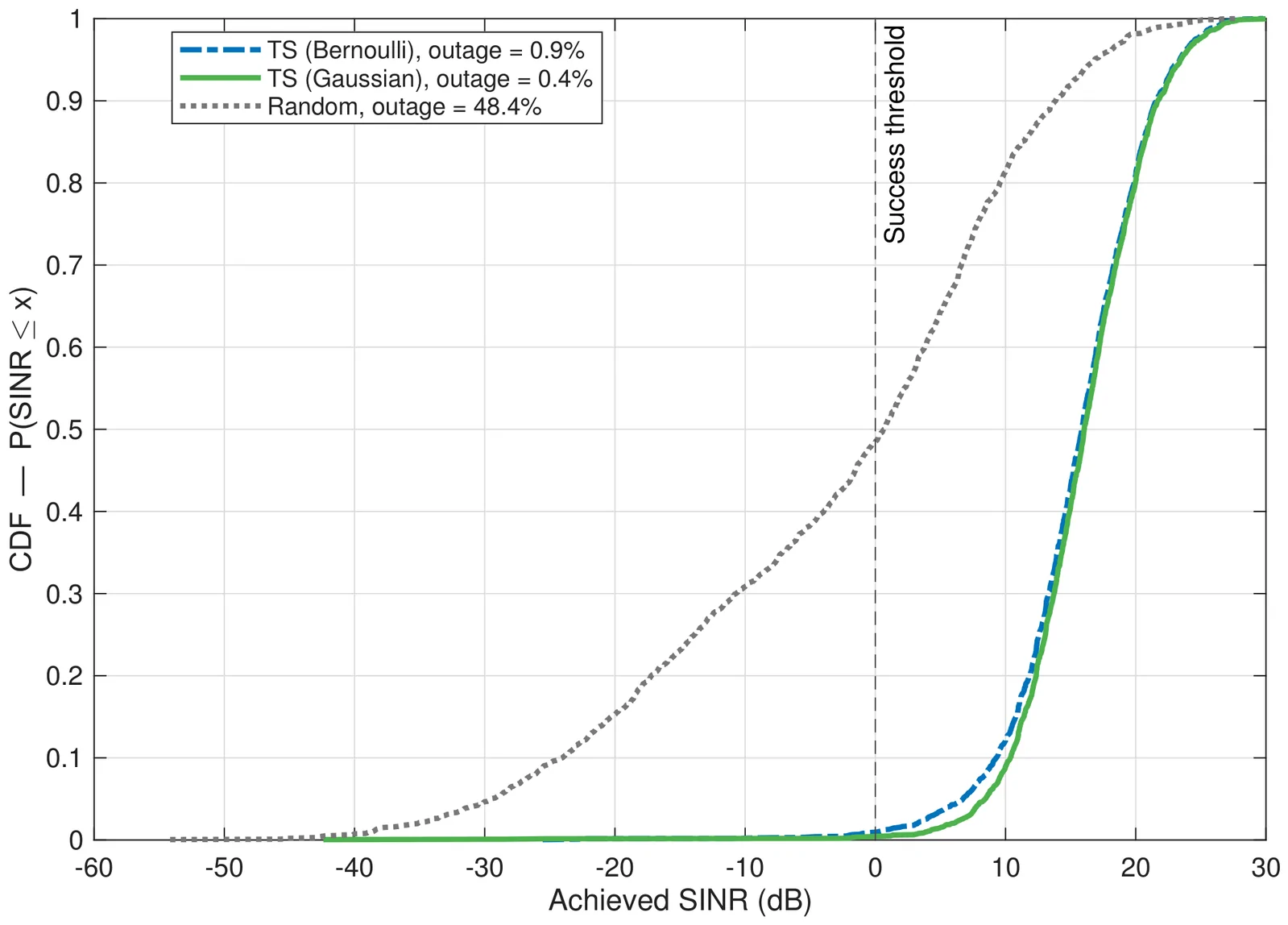
Empirical CDF of achieved SINR per algorithm.

Figure 6 shows the empirical CDF of the achieved SINR for each algorithm, together with the associated outage probability, defined as the fraction of rounds in which the selected relay’s SINR fell below the $\gamma_{\mathrm{th}}=0$ dB usability threshold. In the figure, the outage probability is represented both by the height of each curve at the dashed threshold line and numerically in each legend entry. From an operational standpoint, this is one of the most consequential metrics in this study: an outage represents a scheduling round in which the D2D link selected by the algorithm was, in fact, unusable, regardless of the algorithm’s longer-run average behavior. Both TS variants achieve a low outage probability, with outages almost entirely concentrated in the initial exploration phase during the first few hundred rounds, as visible in Figure 2, Figure 3 and Figure 4. After this initial phase, both algorithms reliably select relays whose SINR distributions remain well above the threshold. Random selection, in contrast, incurs outages throughout the entire run because it has no mechanism for learning to avoid the majority of candidate relays, particularly the NLoS relays. These relays exhibit heavier shadow-fading tails and generally higher path loss, resulting in a substantial fraction of their SINR distribution falling below the threshold, even under favorable fading realizations.

Table 2 summarizes the results at T=2000. The average per-round regret of random selection equals the full gap between the oracle and the mean over relays and never decreases, whereas that of TS-Bernoulli and TS-Gaussian falls to about 0.15 and 0.03 bits/s/Hz, i.e., cumulative regret about 24× and 120× below random.

## 8. Conclusions

This paper studied D2D relay selection at 28 GHz under a 3GPP TR 38.901 UMi-SC channel model as an online learning problem, using two TS variants with distinct objectives: a Beta-Bernoulli model maximizing link-success probability and a Gaussian model maximizing spectral efficiency. Evaluated with shared channel realizations (N=10, T=2000), both variants identified the best relay within about 100–150 rounds and directed more than 94% of their selections to it. Compared with random selection, they raised the mean spectral efficiency by about 3×, raised the link-success rate from 51.6% to above 99%, and reduced the outage probability from 48.4% to 0.9% (TS-Bernoulli) and 0.4% (TS-Gaussian). The two variants performed similarly here because the reward and success oracles coincide in the simulated topology; the Gaussian variant learned faster because the binary signal saturates well above the threshold. Limitations are the stationarity of the large-scale state during learning, the use of a single topology and a random-selection baseline only, and the absence of an informed prior. Future work includes non-stationary and contextual variants, additional baselines, and topologies where the two oracles differ.

## Figures and Tables

**Table 1:** Duration of the 100–150-round learning phase.

Round =	Round Duration	100–150 Rounds
One slot, μ=3 (120 kHz SCS)	0.125 ms	12.5 ms to 18.75 ms
One slot, μ=2 (60 kHz SCS)	0.25 ms	25 ms to 37.5 ms
One radio frame (one report/frame)	10 ms	1 s to 1.5 s
One 20 ms reporting period	20 ms	2 s to 3 s
One 100 ms discovery/reporting period	100 ms	10 s to 15 s

**Table 2:** Summary at T=2000 (N=10). Values marked ≈ are read from Figure 2, Figure 3, Figure 4 and Figure 5.

	TS-Bernoulli	TS-Gaussian	Random
Final mean spectral efficiency (bits/s/Hz)	≈5.25	≈5.4	≈1.75
Cumulative regret	≈300	≈60	≈7300
Link-success rate (%)	99.1	99.6	51.6
Outage probability (%)	0.9	0.4	48.4
Share of selections on best relay (%)	≈94.5	≈99	≈10

## Data Availability

The simulation results and configuration details supporting the findings of this study are available from the corresponding author upon reasonable request.
